# Uncovering the Signaling Pathway behind Extracellular Guanine-Induced Activation of NO System: New Perspectives in Memory-Related Disorders

**DOI:** 10.3389/fphar.2018.00110

**Published:** 2018-02-21

**Authors:** Mariachiara Zuccarini, Patricia Giuliani, Monica Frinchi, Giuseppa Mudò, Rosa Maria Serio, Natale Belluardo, Silvana Buccella, Marzia Carluccio, Daniele F. Condorelli, Francesco Caciagli, Renata Ciccarelli, Patrizia Di Iorio

**Affiliations:** ^1^Department of Medical, Oral and Biotechnological Sciences, Università degli Studi “G. d’Annunzio” Chieti-Pescara, Chieti, Italy; ^2^Aging Research Center, Ce.S.I., “G. d’Annunzio” University Foundation, Chieti, Italy; ^3^Department of Experimental Biomedicine and Clinical Neurosciences, University of Palermo, Palermo, Italy; ^4^Department of Biological, Chemical and Pharmaceutical Sciences and Technologies (STEBICEF), University of Palermo, Palermo, Italy; ^5^Department of Bio-Medical Sciences, University of Catania, Catania, Italy

**Keywords:** guanine, L-NAME, nitric oxide, cGMP, ERK, SH-SY5Y cell line

## Abstract

Mounting evidence suggests that the guanine-based purines stand out as key player in cell metabolism and in several models of neurodegenerative disorders, such as Parkinson’s and Alzheimer’s diseases. Guanosine (GUO) and guanine (GUA) are extracellular signaling molecules derived from the breakdown of the correspondent nucleotide, GTP, and their intracellular and extracellular levels are regulated by the fine-tuned activity of two major enzymes, purine nucleoside phosphorylase (PNP) and guanine deaminase (GDA). Noteworthy, GUO and GUA, seem to play opposite roles in the modulation of cognitive functions, such as learning and memory. Indeed GUO, despite exerting neuroprotective, anti-apoptotic and neurotrophic effects, causes a decay of cognitive activities, whereas GUA administration in rats results in working memory improvement (prevented by L-NAME pre-treatment). This study was designed to investigate, in a model of SH-SY5Y neuroblastoma cell line, the signal transduction pathway activated by extracellular GUA. Altogether, our results showed that: (i) in addition to an enhanced phosphorylation of ASK1, p38 and JNK, likely linked to a non-massive and transient ROS production, the PKB/NO/sGC/cGMP/PKG/ERK cascade seems to be the main signaling pathway elicited by extracellular GUA; (ii) the activation of this pathway occurs in a pertussis-toxin sensitive manner, thus suggesting the involvement of a putative G protein coupled receptor; (iii) the GUA-induced NO production, strongly reduced by cell pre-treatment with L-NAME, is negatively modulated by the EPAC-cAMP-CaMKII pathway, which causes the over-expression of GDA that, in turn, reduces the levels of GUA. These molecular mechanisms activated by GUA may be useful to support our previous observation showing that GUA improves learning and memory functions through the stimulation of NO signaling pathway, and underscore the therapeutic potential of oral administration of guanine for treating memory-related disorders.

## Introduction

Guanine-based purines are known to play crucial role in the modulation of neurotransmission and neuropathologies (Ciccarelli et al., 2001; Boison, 2011; Bettio et al., 2016; Di Liberto et al., 2016). In particular, the purine nucleoside Guanosine (GUO), which is mostly released from astrocytes under pathological conditions (i.e., hypoxic or hypoglycemic stress), is thought to exert both neurotrophic and neuroprotective effects (Di Iorio et al., 2001, 2004; Giuliani et al., 2012, 2015; Lanznaster et al., 2016); indeed, it oversees neuronal development and synaptic activity, and protects neuronal and glial cells against oxidative stress and excitotoxicity (Neary, 1996; Schmidt et al., 2007; Tarozzi et al., 2010; Quincozes-Santos et al., 2014; Bellaver et al., 2015; Ribeiro, 2016; Thomaz et al., 2016). Furthermore, in rats, GUO administration during pre-training displays amnesic effect on inhibitory avoidance task (Roesler et al., 2000; Vinadé et al., 2004; Saute et al., 2006). At present, much less is known about the effects that Guanine (GUA) exerts in the central nervous system. Intracellular GUA derives from guanosine triphosphate (GTP) breakdown and represents the starting point of reactions deputed to maintain intracellular levels of GTP (purine salvage pathway). When intracellular levels of GUA are excessive, it may be transported outside the cells by specific transmembrane nucleobases transporters, although most of the extracellular GUA derives from the breakdown of the released GTP and it is generated by GUO in a reaction catalyzed by the purine nucleoside phosphorylase (PNP) (Rathbone et al., 2008; Giuliani et al., 2016, 2017; Peña-Altamira et al., 2017). On the contrary, GUA degradation to xanthine (Xan) is mediated by Guanine deaminase (GDA) or cypin (Miyamoto et al., 1982), which has been regarded as one of the “intrinsic factors” that regulate dendrite morphology together with the small GTPases RhoA, Rac1, the β-catenin (Yu and Malenka, 2004), PSD-95 (Charych et al., 2006) and the calcium/calmodulin-dependent protein kinase II (CAMKII) (Fink et al., 2003). CaMKII is a synaptic signaling molecule that plays a crucial role during long-term memory formation (Lucchesi et al., 2011) and its endogenous inhibitors CaMK2N1 and CaMK2N2 are highly expressed during memory consolidation (Lepicard et al., 2006). Cyclic AMP-CREB axis is implicated in learning and memory processes and has been shown to activate CaMKII.

In a previous work (Giuliani et al., 2012), we reported the effects of GUO and GUA on learning and memory in a model of passive avoidance task in rats. In that study, the oral administration of GUO exerted amnesic activity on inhibitory avoidance task and was unable to prevent the amnesic effect caused by *N*-omega-nitro-l-arginine methyl ester (L-NAME), a non-specific NOS inhibitor known to reduce the capability of treated animals to acquire or retain information in several learning tasks. Conversely, the administration of GUA counteracted the L-NAME-mediated amnesic effects, by increasing the step-through latency either when it was given in the learning phase or during the memory consolidation phase.

In addition to GUO and GUA, another guanine-based purine has been correlated to changes in memory processes, namely cyclic guanosine monophosphate (cGMP), which exerts memory-enhancing effect through the modulation of NMDA receptors and the glutamate-nitric oxide (NO) pathway (Cabrera-Pastor et al., 2016) or via NOS-soluble guanylyl cyclase (sGC)-cGMP- protein kinase G (PKG) pathway (Friebe and Koesling, 2003; Boess et al., 2004; Masood et al., 2009; Bollen et al., 2014; Lueptow et al., 2015). Noteworthy, there is a large body of evidence confirming the existence of a cross-talk between NO and ERK signaling pathways during memory formation and learning processes (Moosavi et al., 2014). Indeed, it has been shown that ERK represents a crucial downstream mediator of NO in the brain (Chien et al., 2008) and that the blockage of NO-cGMP-PKG prevents the activation of ERK mediated by high-frequency stimulation-(HFS) (Ping and Schafe, 2010).

Based on the above mentioned mnesic effects elicited, *in vivo*, by GUA, and several findings showing that NO-cGMP-PKG-ERK signaling pathway is positively correlated with enhancement of memory formation (Adams and Sweatt, 2002; Davis and Laroche, 2006; Giovannini, 2006; Philips et al., 2007; Chien et al., 2008; Adaikkan and Rosenblum, 2012), in this study we aimed to:

(a) verify, by using human neuroblastoma cell line SH-SY5Y, if cGMP and NO-PKG-ERK signaling pathway resulted to be activated upon cell exposure to GUA;
(b) assess whether the activation of this signaling pathway may involve the extracellular GUA interaction with a new putative receptor.

## Materials and Methods

### Materials and Chemicals

The human neuroblastoma cell line SH-SY5Y was purchased from European Collection of Authenticated Cell Culture (ECACC, Salisbury, United Kingdom); Guanine, Guanosine, Nutrient Mixture F-12 Ham, Minimum Essential Medium Eagle (MEM), Non-Essential Amino Acids (NEAA), L-Glutamine, Trypsin-EDTA, Pertussis toxin from Bordetella pertussis (PTX), 3-Isobutyl-1-methylxanthine (IBMX), 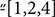 Oxadiazolo[4,3-a]quinoxalin-1-one (ODQ), 8-(4-Chloro-phenylthio)-2′-*O*-methyladenosine 3′,5′-cyclic monophosphate monosodium hydrate (8-pCPT-2′-O-Me-cAMP), N-[2-[N-(4-Chlorocinnamyl)-*N*-methylaminomethyl]phenyl]-N-(2-hydroxyethyl)-4-methoxybenzenesulfonamide phosphate salt, N-[2-[[[3-(4′-Chlorophenyl)-2-propenyl]methylamino]methyl]phenyl]-*N*-(2-hydroxyethyl)-4′-methoxybenzenesulfonamide phosphate salt (KN-93), Propentofylline, S-(4-Nitrobenzyl)-6-thioinosine (NBTI), 2′,7′-Dichlorofluorescin diacetate (H2DCF-DA), dimethylsulfoxide (DMSO), Ionomycin, trypsin/EDTA, EDTA, EGTA, HEPES, Phosphate Buffer Solution (PBS), dithiothreitol (DTT), NADPH, calmodulin, CaCl2, tetrahydrobiopterin and the cationic exchange resin Dowex AG50WX-8, *N*-(1-naphthylethylenediamine) dihydrochloride, were purchased from Sigma (Milan, Italy); NG-Nitro-L-arginine methyl ester hydrochloride (L-NAME), MSC 20329644, GF109203X, 10-DEBC hydrochloride, Dipyridamole and LY 294002 hydrochloride were purchased from Tocris (Milan, Italy); Penicillin-streptomycin and Heat-inactivated fetal bovine serum (FBS) were purchased from Gibco^®^ (Thermo Fischer Scientific, Monza, Italy); Phospho-ASK1, Phospho-p38 MAPK, Phospho-SAPK/JNK, Phospho-PKC (pan), Phospho-Akt, Phospho-p44/42 MAPK (Erk1/2), β-Actin, secondary anti-rabbit IgG HRP-linked antibody were purchased from Cell Signaling Technology (Cell Signaling, Leiden, Netherlands); PNP and Guanase Deaminase antibodies were purchased from Novus Biologicals (Space Import-Export, Milan, Italy).

### Cell Culture

The human neuroblastoma cells, SH-SY5Y, were cultured in 75 cm^2^ flasks in a 1:1 mixture of F-12 nutrient mixture (Ham 12) and Eagle’s MEM (EBSS) supplemented with 2 mM Glutamine, 1% Non-Essential Amino Acids (NEAA), 15% Foetal Bovine Serum (FBS) and 100 units/mL penicillin and 100 μg/mL streptomycin and maintained at 37°C in 5% CO2, humified air.

For the evaluation of PNP and GDA release, cell medium was removed and replaced by serum free-medium and maintained in humified atmosphere, 5% CO2, 37°C. At the end of the experiment, aliquots (2 mL) of the culture medium were collected, placed in suitable devices (Amikon Ultra 2 mL, cutoff 10 K, Merck Millipore, Germany) and centrifuged following the manufacturer’s instruction, in order to concentrate culture media containing the enzymes.

For the evaluation of purine release, cell medium was removed and replaced with Krebs-HEPES buffer (15 m M HEPES, pH 7.4, 120 mM NaCl, 4 mM KCl, 1.2 mM MgSO_4_, 1 mM CaCl_2_), and 10 mM D-glucose oxygenated (95% O2/5% CO2). After 30 min, the cells were incubated for further 30 min with the same buffer containing 2.5 μM GUO combined with 0.675 μM of [3H]GUO (specific activity 5.3 Ci/mmol; Movarek Biochemicals). At the end of this incubation period, cells were washed twice with unlabeled Krebs-HEPES buffer and maintained in this medium in standard condition (37°C, 5% CO2). When used, purine uptake inhibitors were added to Krebs-HEPES just after the incubation with labeled GUO. At the end of the experiment, an aliquot of the culture medium was collected and immediately heat-inactivated for 5 min at 70°C to avoid any further enzymatic degradation of the released purine. Samples were, then, centrifuged, filtered with 0.2 μm filters (Millipore, Vimodrone, Italy) and stored at 80°C before HPLC analysis.

For Immunoblot assays, SH-SY5Y cells were subcultured in 100 × 20-mm Petri Dishes (BD Falcon) at a seeding density of 2 × 10^5^ per dish (for each sample two dishes were pulled together) and grown until 80% confluence. Before all experiments, cells were starved for 24 h in medium containing 0.1% FBS.

### HPLC Method for the Evaluation of Purine Levels in the Extracellular Milieu

According to the method previously described (Giuliani et al., 2012, 2017), purines were measured by an Agilent 1100 series HPLC system (Agilent Technologies, Santa Clara, CA, United States), by using, for the separation of the compounds, a reverse phase analytical column (LiChrospher 100 RP-18 5 μm in LiChroCART 125-4, Merck) and a 15-min linear gradient [from 100% of buffer A (60 mM KH_2_PO_4_ and 5 mM tetrabutylammonium phosphate, pH 6.0) to 100% solvent B (30% methanol plus 70% buffer A)] at a flow rate of 1.5 mL/min. The detection of unlabeled compounds was achieved using a Diode Array Detector (Agilent Technologies, Santa Clara, CA, United States) with wavelength set at 254 nm for all the substances except Uric Acid (UAc), which was 290 nm. Released purines were identified and quantified by comparison with pure external standards. Since many purine compounds are present in the extracellular milieu at concentrations below the UV detection limit, the HPLC system was equipped with an online radiochemical detector (FLO-ONE 500 TR, Packard Instruments) for the concurrent measurement of radiolabeled purine present in the outflow from the Diode Array Detector, in order to improve the sensibility of the analysis. Thus, the HPLC effluent was mixed with the liquid scintillation cocktail (Ultima-FloM, Perkin-Elmer) at a flow ratio of 2:1 and passed through a 500 μL detector flow cell. Radio-chromatograms were integrated and each radioactive peak was quantified.

### Measurement of Enzyme Activity in the Extracellular Milieu

PNP activity was measured in an assay buffer containing 50 mM HEPES, pH 7.0, 50 mM inorganic phosphate (Pi), used as co-substrate, and 200 μM GUO used as substrate, whereas the mixture used to evaluate GDA activity consisted of 100 mM TrisHCl pH 8 plus 200 μM GUA used as substrate. These enzymatic reactions were started by adding aliquot of the concentrated extracellular culture medium. The mixtures were then incubated by shaking at 37°C for 15 min, to evaluate PNP activity, and for 60 min, to determine GDA activity. The reactions were stopped by heating the mixture at 70°C for 5 min and the precipitated proteins were removed by centrifugation. The enzyme activity was determined by quantifying the rate of conversion of GUO to GUA, for PNP, or the conversion of GUA to XAN for GDA, using the HPLCmethod previously described (Giuliani et al., 2016). In this case, the Agilent HPLC was equipped with a thermostated column compartment, a diode array detector, and a fluorescence detector (Agilent Technologies). Briefly, separation was achieved using a Phenomenex Kinetex pentafluorophenyl analytical column (5 μm pore size, 100 Å particle size, 250 × 4.6 mm; Phenomenex INC) at 35°C. Separation was carried out with a 15-min non-linear gradient elution (flow rate 1 mL/min) using a mobile phase composed of 0.1% (v/v) formic acid in water (solution A) and methanol (solution B). The fluorescent GUO and GUA were monitored at an excitation wavelength of 260 nm and an emission wavelength of 375 nm, whereas for the non-fluorescent compounds, i.e., XAN and UAc, the UV detector was set at 254 and 290 nm respectively. Allsubstances were identified and quantified by comparison with pure external standards. Enzyme activity was expressed as Unit (U) present in the total medium, being 1 U of enzyme the amount of enzyme that converts 1 μmole of substrateinto product per min.

### Cell Viability Assay

Cell death was monitored by using the CytoTox-96 assay (Promega Italia, Milan, Italy) that allows to evaluate the lactate dehydrogenase (LDH) activity. The assay is based on a 30-min coupled enzymatic assay, catalyzed by released LDH, which results in conversion of a tetrazolium salt, 2-p-(iodophenyl)-3-(p-nitrophenyl)-5-phenyltetrazolium chloride (INT), into a red formazan product. SH-SY5Y cells were seeded in 96-well plates at 5 × 10^3^ cells/well of confluence and incubated for 2 days. For all samples, the cell culture medium was replaced with Krebs-HEPES buffer (15 mM HEPES, pH 7.4, 120 mM NaCl, 4 mM KCl, 1.2 mM MgSO_4_, 1 mM CaCl_2_) with or without 50 μM GUA (0–12 h). At the end of exposure, the Lysis solution was added for 45 min to control wells for the determination of maximum LDH release. Afterward, 50 μL of collected media were transferred to a fresh 96-well (enzymatic assay) plate, together with 50 μL of Substrate buffer containing 0.7 mM *p*-iodonitrotetrazolium Violet, 50 mM L-lactic acid, 0.3 mM phenazine methoxysulfate, 0.4 mM NAD and 0.2 M Tris-HCl pH 8.0. Finally, the plate was protected from light and incubated for 30 min at RT. The absorbance was recorded at 490 nm of wavelength in a microplate reader after adding the Stop Solution. LDH activity was expressed as the proportion of LDH released into the culture medium compared to the total amount of LDH present in cells lysates and calculated as follows: (medium absorbance value – white absorbance value)/(medium absorbance + lysate absorbance) × 100.

### Immunoblot

SH-SY5Y cells were seeded overnight onto 100 mm Petri Dishes (BD Falcon) at 2.0 × 10^5^ cells/dish in 6 mL of 1:1 mixture of F-12 nutrient mixture (Ham 12) and Eagle’s MEM (EBSS) supplemented with 2 mM Glutamine, 1% Non Essential Amino Acids (NEAA), 15% Foetal Bovine Serum (FBS) and 100 units/mL penicillin and 100 μg/mL streptomycin. After 24-h starvation, cells were submitted to different treatments in MEM supplemented with 0.5% FBS and 1% Penicillin/Streptomycin. After treatment, cells were washed twice with ice cold 1× PBS (Sigma–Aldrich), lysed with RIPA Buffer (Sigma–Aldrich) containing 150 mM NaCl, 10 mM EDTA, 1% NP40, 0.5% deoxycholic acid, 0.1% SDS, and 50 mM Tris, pH 7.5, supplemented with 1% Protease Inhibitor Cocktail (Sigma–Aldrich), scraped off, pulled, and clarified by centrifugation at 12.500 × *g* for 20 min, 4°C. Before performing Immunoblot, a sample buffer (5× Laemmli buffer with 10% mercaptoethanol) was added to melted lysates 1:4. Protein concentrations were obtained using the Bio-Rad Protein Assay (Bio-Rad Laboratories, Hercules, CA, United States) based on the Bradford method. An equal amount of 50–70 μg of protein was resolved by 10% sodium dodecyl sulfate–polyacrylamide gel electrophoresis (SDS-PAGE). The resolved proteins were transferred onto a nitrocellulose membrane and then incubated with blocking buffer 1× TBS containing 0.1% Tween-20 (TBST) and 3% BSA or 5% non-fat dry milk for 2 h, RT, and subsequently probed with specific primary antibody at 4°C, overnight. After washing with TBST, the membrane was further probed with corresponding horseradish peroxidase (HRP)-conjugated secondary antibodies at RT for 1 h. Membranes were finally washed, before subjecting them to ECL Plus Immunoblot Detection Reagent (Amersham, GE Healthcare). The immunoreactive bands were visualized under a chemiluminescence detection system (UVItec, Cambridge, United Kingdom). Band intensity data were obtained using Quantity One software (Bio-Rad Laboratories). Blotting membranes were stripped and re-probed with anti-actin antibody as equal loading control. Estimates of phosphorylated proteins are presented as densitometric ratios, normalized to the corresponding total protein content. Apart from PNP antibody (1:500), all primary antibodies [Phospho-ASK1 (Ser83), Phospho-p38 MAPK (Thr180/Tyr182), Phospho-SAPK/JNK (Thr183/Tyr185), Phospho-PKC (Ser660), Phospho-Akt (Thr450), Phospho-p44/42 MAPK (Erk1/2) (Thr202/Tyr204), Guanase Deaminase, β-Actin] were diluted 1:1000 in 3% BSA/1× TBS/0.1% Tween 20 or 2.5% non-fat dry milk/1× TBS/0.1% Tween 20. The secondary antibody was used at 1:2500 dilution in 3% BSA/1× TBS/0.1% Tween 20 or 2.5% non-fat dry milk/1× TBS/0.1% Tween 20.

### Measurement of Cellular Reactive Oxygen Species (ROS)

The amount of intracellular reactive oxygen species (ROS) was measured by using the probe H_2_DCF-DA (Ha et al., 1997), which diffuses into the cells and is oxidized to the green fluorescent compound 2′,7′-dichlorofluorescein (DCF) upon reaction with intracellular hydrogen peroxide or low-molecular-weight hydroperoxides. Cells were seeded at 1 × 10^6^ cells/well in 6-well culture plates and incubated overnight. After exposure to different concentration of GUA for 30 min, cells were incubated with 5 μM H_2_DCF-DA for 30 min, in the dark, at 37°C. At the end of incubation, the cells were washed with PBS and fluorescence was measured at an excitation wavelength of 480 nm and an emission wavelength of 540 nm in a fluorescence microplate reader (Thermo Fischer Scientific, Monza, Italy). ROS production was determined by analyzing DCF fluorescence normalized for total protein content. The fluorescence intensity was proportional to the amount of ROS produced by cells.

### Determination of Nitric Oxide Synthase (NOS) Activity

Nitric oxide synthase activity was measured from the conversion of L-[3 H]-arginine to L-[3 H]-citrulline based on the method of Bredt et al. (1991) with modifications. SH-SY5Y cells were grown overnight in 6-well plates. After 24-h starvation, cells were exposed for 30 min to 50 μM GUA, 5 μM L-NAME or 2 μM Ionomycin, the latter used as positive control. When used in combination, L-NAME was administered 15 min before cell exposure to GUA or Ionomycin. Thereafter, cells were washed three times with ice-cold 1X PBS, scraped in 1X PBS containing 1 mM EDTA, and centrifuged for 10 min at 1200 *g*. The pellets were resuspended in a reaction buffer containing 50 mM Hepes, 1 mM EDTA, 1mM DTT (pH 7.2) and sonicated on ice with two 10 s bursts. The reaction was started by addition to samples of reaction mixture [1 mM NADPH, 1 nmol/l calmodulin, 1.25 mM CaCl2, 3 μM tetrahydrobiopterin, 2.5 μCi/μl of L-[3H]arginine (Perkin Elmer, Boston, MA, United States, specific activity 42.6 Ci/mmol), unlabelled arginine]. After an incubation of 15 min at 37°C, the assay was stopped by adding 20 mM Hepes-Na containing 2 mM EDTA and 2 mM EGTA (pH 5.5), and the reaction mixture was applied to 2-ml columns of Dowex AG50WX-8 (Na+ form), which were eluted with 4 ml of water. The radioactivity corresponding to the [3H]-citrulline was measured by liquid scintillation analyzer (Tris-Carb 2100 TR, Perkin Elmer) and normalized for extract protein content determined with Bradford method. NOS activity was expressed as pmoles citrulline/min/mg cell protein.

### Statistical Analysis

Data are represented as means ± standard error of mean (SEM). Comparisons among experimental groups were performed by Student *t*-test or by two-way ANOVA followed by Sidak’s multiple comparisons test using GraphPad Prism 6.01 (San Diego, CA, United States), as indicated. Statistical difference was accepted when *P* < 0.05. All experiments were performed at least three times.

## Results

### The Levels of Guanine-Based Purines in SH-SY5Y Culture Media Are Controlled by Specific Nucleobase and Nucleoside Transporters and by the Presence of Purine-Converting Enzymes

A hallmark of several neurodegenerative diseases is the activation of neuronal and glial cells and the following induction of oxidative stress and neuronal toxicity. In the attempt to investigate neuronal response to GUA exposure, we used SH-SY5Y, a human derived neuroblastoma cell, which has been often used as a model to study molecular mechanisms associated to ROS production, apoptosis and amyloid-β-induced neuronal toxicity in Alzheimer’s disease (Tarozzi et al., 2010; Ali-Rahmani et al., 2014; Puangmalai et al., 2017; Modi et al., 2017).

**FIGURE 1:**
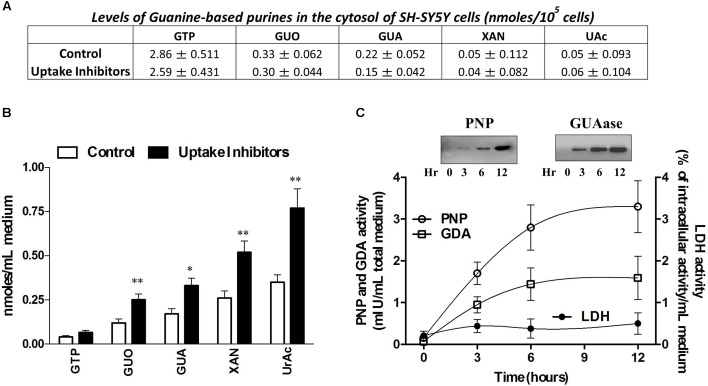
SH-SY5Y neuroblastoma cells release guanine-based purines, PNP and GDA in the culture medium. **(A)** HPLC analysis of intracellular levels of Guanine-based purines in SH-SY5Y cells. **(B)** HPLC analysis of extracellular levels of guanine-based purines at rest (control) and in the presence of the inhibitors of cell uptake. SH-SY5Y were incubated with 2.5 μM guanosine (GUO) combined with 0.675 μM of [3H]GUO, the latter used as tracer. Values are expressed as nmoles/mL of culture medium, and represent the mean ± SEM of five independent experiments. ^∗^*p* < 0.05; ^∗∗^*p* < 0.01: statistical significance versus untreated cells (control) (Student’s *t*-test). **(C)** Evaluation of the presence and activity of purine nucleoside phosphorylase (PNP) and guanine deaminase (GDA) in the culture medium of SH-SY5Y cells. (C1) Representative Immunoblots of PNP and GDA expression in SH-SY5Y culture medium. After 24 h incubation, SH-SY5Y cell culture medium was collected after 3, 6, and 12 h, concentrated and analyzed for PNP and GDA expression. (C2) HPLC analysis of PNP and GDA activity evaluated up to 12 h. PNP activity was assayed by using 200 μM guanosine (GUO) as substrate plus 50 mM Pi as co-substrate for 15 min at 37°C, whereas GDA activity was measured by using 100 mM TrisHCl pH 8 plus 200 μM GUA as substrate for 60 min at 37°C. Values are expressed as milli-International Units (mIU) of enzyme per total culture medium and represent the mean ± SEM of three independent experiments, run in duplicate. (C3) Evaluation of cell viability of SH-SY5Y cells by LDH assay. Values are expressed as the percentage of the intracellular LDH activity that was determined after cell lysis, and represent the mean ± SEM of five different experiments.

We firstly measured both the intracellular and extracellular levels of purines in cultured SH-SY5Y cells, in resting conditions. In the SH-SH5Y lysates, the levels of Guanine-based nucleotides prevailed over the correspondent nucleobases and the inhibition of the uptake, performed by treating cell culture with a cocktail of inhibitors of nucleoside and nucleobase transmembrane transporters (100 μM propentofylline, 10 μM NBTI, 10 μM dypiridamole) did not caused any significant effect (**Figure 1A**). An opposite trend was observed in the culture medium, wherein the uptake inhibitors significantly increased extracellular levels of GUA, Xan and UAc [two-way ANOVA analysis and Sidak’s multiple comparisons test: *P* < 0,005] (**Figure 1B**). This suggested that the levels of extracellular guanine-based purines depended both on the balance between nucleotide release and nucleoside uptake and on the presence, in the culture medium, of extracellular purine-converting enzymes. Indeed, by using Immunoblot analysis and HPLC for the measurement of the enzyme activities (Giuliani et al., 2017), we confirmed the presence in the culture medium of PNP and GDA (**Figure 1C**). Both the enzymes tended to accumulate in the culture medium over the time (from 2 up to 12 h) following a similar trend even though at different levels (PNP amount was about 7–10 fold higher than GDA). This event was not associated with significant cell damage or death, as demonstrated by the constant and minor presence of LDH, measured during the same period in the culture media (**Figure 1C**).

### Guanine Increases ASK1, p38, JNK, PKC and PKB Phosphorylation and This Effect Is Prevented, Except for PKB, by Cell Treatment with a Cocktail of Inhibitors of the Uptake

**FIGURE 2:**
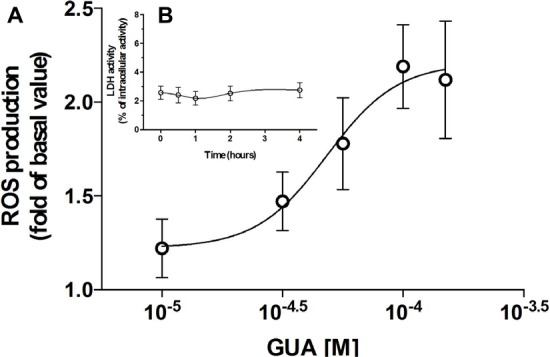
Guanine (GUA) induces ROS production in SH-SY5Y neuroblastoma cells in a dose-dependent manner. **(A)** SH-SY5Y cells were incubated for 30 min with different concentrations of GUA (0–100 μM). The levels of ROS are presented as folds of control and represent the mean ± SEM of five different experiments. **(B)** Evaluation of cell viability of SH-SY5Y cells exposed to 50 μM GUA up to 4 h. Values are expressed as the percentage of the intracellular LDH activity that was determined after cell lysis and represent the mean ± SEM of five different experiments.

**FIGURE 3:**
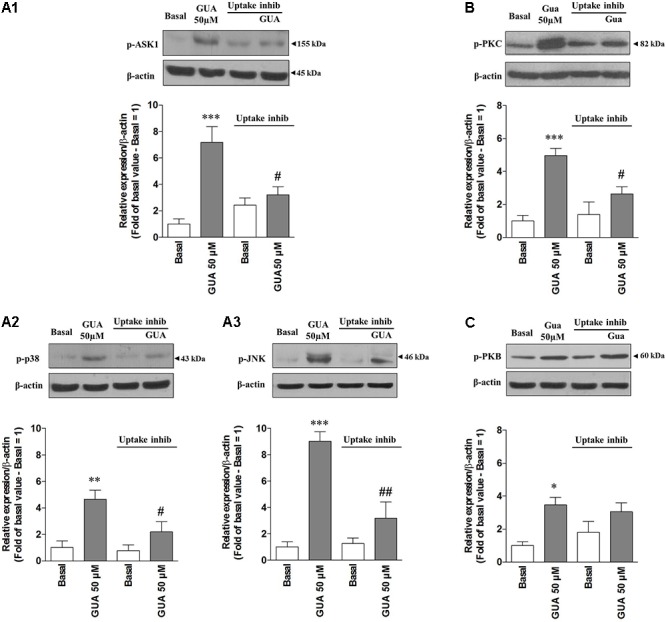
Guanine induces ASK1, p38, JNK, PKC and PKB phosphorylation in SH-SY5Y neuroblastoma cells. SH-SY5Y cells were cultured for 24 h and incubated for 10 min with 50 μM GUA, in the absence or presence of a cocktail of inhibitors of both nucleoside and nucleobase transmembrane transporters (100 μM Propentofylline, 10 μM NBTI, 10 μM Dypiridamole). Representative Immunoblot analysis of **(A1)** p-ASK1 (Thr845), **(A2)** p-p38, **(A3)** p-JNK, **(B)** PKC, **(C)** p-PKB, with the respective β-actin as loading control, and the correspondent quantitative data of densitometric analysis. Each column represents the mean ± SEM of four independent experiments, and it is expressed as relative protein expression normalized to β-actin. Student’s *t*-test: ^∗^*P* < 0.05, ^∗∗^*P* < 0.01, ^∗∗∗^*P* < 0.001, vs. untreated cells (Basal); #*P* < 0.05, ##*P* < 0.01, vs. GUA-treated cells.

Cell exposure to exogenous GUA, for 30 min, elicited a non-massive and dose-dependent production of ROS. This occurred without any significant collateral LDH production (**Figure 2**). A similar effect has been found in different glial cells (astrocytes or microglial cells, data not shown), wherein ROS production seemed to be even greater than that elicited in SH-SY5Y cells. The ROS production has been often associated with an increased phosphorylation of ASK1, p38 and JNK (Shen and Liu, 2006; Jiang et al., 2017) and it has been reported that the inhibition of c-Jun N-terminal Kinase (JNK) in SH-SY5Y cells, prevented 6-Hydroxydopamine-induced ROS production and toxicity (Feng et al., 2013). Based on this evidence, we investigated which signaling transduction pathways were involved in GUA-induced effect. SH-SY5Y cells were exposed to 50 μM GUA, the half maximal effective concentration, for 10 min, and the expression of p-ASK1, p-p38, p-JNK, PKC and PKB was analyzed by Immunoblot. The short-term exposure of cells to GUA significantly increased the phosphorylation of all the mentioned kinases. The two-way ANOVA analysis of their expression showed an effect of cell exposure to GUA [*F*_(1,12)_ = 27.53, *P* = 0.0002 for p-ASK1; *F*_(1,12)_ = 16.89, *P* = 0.0014 for p-38; *F*_(1,12)_ = 41.35, *P* = 0.0001 for p-JNK; *F*_(1,12)_ = 16.09, *P* = 0.0017 for PKC and *F*_(1,12)_ = 27.07, *P* = 0.0002 for PKB] (**Figures 3A,B**). Cell pre-treatment with the previously mentioned cocktail of inhibitors of both nucleoside and nucleobase transmembrane transporters, 30 min before cell exposure to GUA, did not modify the basal phosphorylation level of all kinases but strongly reduced GUA-induced phosphorylation of ASK1, p38, JNK and PKC, [two-way ANOVA: *F*_(1,12)_ = 4.77, *P* = 0.045 for p-ASK1; *F*_(1,12)_ = 13.07, *P* = 0.0035 for p-JNK; *F*_(1,12)_ = 7.20, *P* = 0.019 for PKC with interaction values between factors of *F*_(1,12)_ = 13.86, *P* = 0.0029; *F*_(1,12)_ = 15.51, *P* = 0.002 and *F*_(1,12)_ = 22.49, *P* = 0.0005, respectively], revealing that the effect of GUA on these pathways was mainly intracellular (**Figures 3A,B**). Conversely, GUA-induced phosphorylation of PKB was not modified, [two-way ANOVA: *F*_(1,12)_ = 0.6131, *P* = 0.4488 and no effect of the interaction between both factors *F*_(1,12)_ = 0.01, *P* = 0.9212], thus suggesting that the activation of this pathway mainly depends on the extracellular activity of GUA (**Figure 3C**).

### PI3K-PKB-ERK Is the Main Signaling Pathway Activated by Extracellular GUA in SH-SY5Y Cells

**FIGURE 4:**
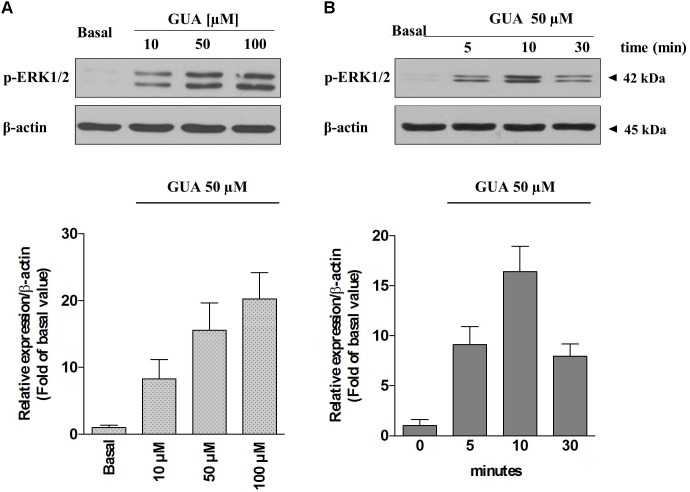
Guanine induces ERK1 phosphorylation in SH-SY5Y neuroblastoma cells in a dose-dependent manner. Representative Immunoblot analysis of ERK1/2 expression in SH-SY5Y cells, with β-actin as loading control, and the correspondent quantitative data of densitometric analysis. **(A)** Cells were cultured for 24 h and exposed for 10 min to different concentrations of GUA (0–100 μM). **(B)** The time course of activation of ERK1/2 induced by GUA. Cells were cultured for 24 h and exposed to 50 μM GUA for different time points (0–30 min). Each column represents the mean ± SEM of at least four independent experiments, and it is expressed as relative protein expression normalized to β-actin.

**FIGURE 5:**
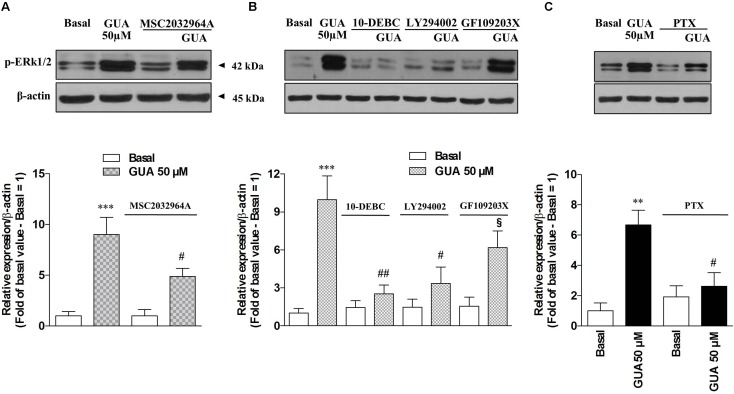
Guanine induces ERK1 phosphorylation in SH-SY5Y neuroblastoma cells via PI3K-PKB-ERK signaling pathway in a PTX-sensitive manner. Representative Immunoblot analysis of ERK1/2 expression in SH-SY5Y cells, with β-actin as loading control, and the correspondent quantitative data of densitometric analysis. Cells were cultured for 24 h and exposed for 10 min **(A,B)** or for 4 h **(C)** to 50 μM GUA, in the absence or presence of **(A)** ASK1 inhibitor, 10 μM MSC2032964A, **(B)** PKC inhibitor (1 μM GF109203X), PKB inhibitor (10 μM 10-DEBC dihydrochloride), PI3K inhibitor (25 μM LY294002), **(C)** 200 ng/mL Pertussis Toxin (PTX.) Each column represents the mean ± SEM of at least four independent experiments, and it is expressed as relative protein expression normalized to β-actin. Student’s *t*-test: ^∗^*P* < 0.05, ^∗∗^*P* < 0.01, ^∗∗∗^*P* < 0.001, vs. untreated cells (Basal); ^#^*P* < 0.05, ^##^*P* < 0.01, vs. GUA-treated cells, §*P* < 0.05, vs. GUA- and GF109203X-treated cells.

Due to the evidence that ERK stands out as a key player in the modulation of several memory processes (Giovannini, 2006) and that, in different experimental models, it represents the downstream effector of ROS-induced phosphorylation of ASK1, p38 and JNK (Lee and Cheong, 2017), we sought to determine the relevance of each of these pathways on GUA-induced effect, by selectively blocking ASK1 or PKC, two upstream kinases in ERK signaling. Immunoblot analysis of p-ERK1/2 expression revealed that GUA elicited a dose-dependent phosphorylation of ERK (10-100 μM), [two-way ANOVA analysis and Sidak’s multiple comparisons test: *P* < 0,005 for GUA 50 and 100 μM] and the time course [two-way ANOVA analysis: *F*_(1,24)_ = 74.57, *P* < 0,0001 with interaction values between factors of *F*_(2,24)_ = 5.053, *P* = 0.0147] showed that the maximum effect of the purine base was achieved in 10 min and decreased after 30 min (**Figure 4**). The inhibition of ASK1 or PKC by cell pre-treatment with 10 μM MSC2032964A or 1 μM GF109203X for 30 min, respectively, did not affect basal ERK phosphorylation, and only in part reduced that induced by GUA [two-way ANOVA: GUA effect *F*_(1,16)_ = 4.543, *P* < 0.0489, ASK1 inhibitor effect *F*_(1,16)_ = 37.66, *P* < 0.0001 with interaction values between factors of *F*_(3,32)_ = 4.449, *P* = 0.0499; GUA effect *F*_(1,16)_ = 3.220, *P* < 0.0917, PKC inhibitor effect *F*_(1,16)_ = 39.35, *P* < 0.0001 with interaction values between factors of *F*_(3,32)_ = 4.504, *P* = 0.0498]. Conversely, the inhibition of PKB by cell pre-treatment with 10 μM 10-DEBC dihydrochloride, as well as the inhibition of the upstream PI3K by 25 μM LY294002, did not modify the basal p-ERK expression and strongly reduced that elicited by GUA [two-way ANOVA: GUA effect *F*_(1,16)_ = 10.84, *P* < 0.0046, 10-DEBC effect *F*_(1,16)_ = 22.53, *P* < 0.0002 with interaction values between factors of *F*_(3,32)_ = 13.82, *P* = 0.0019; GUA effect *F*_(1,16)_ = 6.456, *P* < 0.0218, LY294002 effect *F*_(1,16)_ = 20.12, *P* < 0.0004 with interaction values between factors of *F*_(3,32)_ = 8.564, *P* = 0.0098] (**Figures 5A,B**). This suggests that, in SH-SY5Y cells, PI3-PKB-ERK signaling cascade is likely the main pathway activated by extracellular GUA.

### GUA Activates PKB-ERK Signaling Pathway in a Pertussis-Toxin Sensitive Manner

We, then, inquired whether GUA effect may be ascribed to an eventual interaction with a new putative receptor. For this purpose, SH-SY5Y cells were treated for 4 h with 200 ng/mL Pertussis Toxin (PTX), a specific inhibitor of Gi/Go-proteins, and the expression of p-ERK 1/2 was analyzed by Immunoblot. PTX did not affect basal ERK1/2 phosphorylation but significantly reduced that induced by GUA [two-way ANOVA: *F*_(1,16)_ = 9.795, *P* = 0.0065 with interaction values between factors of *F*_(1,16)_ = 16.09, *P* = 0.0010], thus supporting the involvement of a G_i_ protein-coupled receptor in the effect of GUA (**Figure 5C**).

### Extracellular GUA Stimulates the PI3K-PKB Phosphorylation and NO Production and Activates the Downstream sGC-cGMP-PKG-ERK Pathway

**FIGURE 6:**
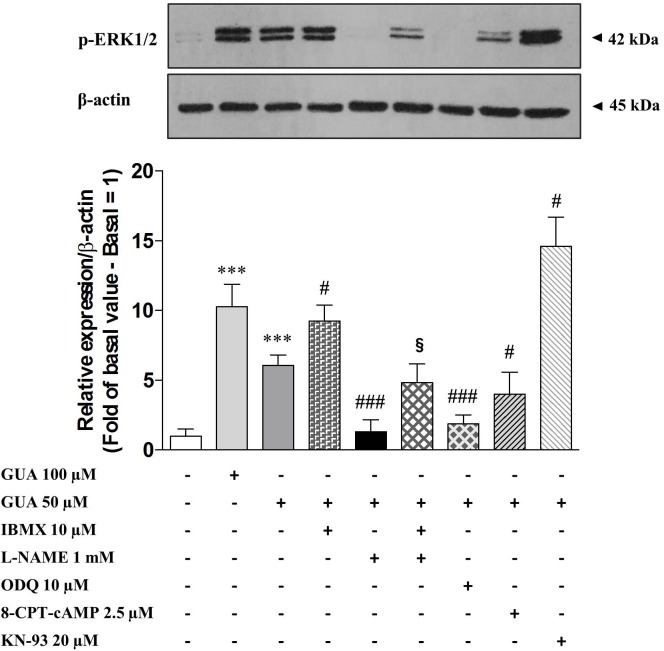
Guanine activates sGC-cGMP-PKG-ERK signaling pathway in SH-SY5Y neuroblastoma cells. Representative Immunoblot analysis of ERK1/2 expression in SH-SY5Y cells, with β-actin as loading control, and the correspondent quantitative data of densitometric analysis. Cells were cultured for 24 h and exposed for 30 min to 100 μM GUA, 50 μM GUA, a phosphodiesterase inhibitor (10 μM IBMX), a NOS inhibitor (1 mM L-NAME), a sGC inhibitor (10 μM ODQ), an EPAC specific cAMP analog (2,5 μM 8-CPT-cAMP), a CaMKII inhibitor (20 μM KN-93). Each column represents the mean ± SEM of at least three independent experiments, and it is expressed as relative protein expression normalized to β-actin. Student’s *t*-test: ^∗∗∗^*P* < 0.001, vs. untreated cells (Basal); ^#^*P* < 0.05, ^###^*P* < 0.001, vs. GUA-treated cells, ^§^*P* < 0.05, vs. GUA- and L-NAME-treated cells.

Nitric oxide (NO) is known to induce ERK phosphorylation via sGC-cGMP-PKG pathway, (Prickaerts et al., 2001; Suvarna and O’Donnell, 2002; Boess et al., 2004; Rutten et al., 2007; Chien et al., 2008; Masood et al., 2009; Xu et al., 2013; Bollen et al., 2014; Matsumoto et al., 2016). Hence, we evaluated the involvement of NO system on GUA-induced ERK phosphorylation, by selectively blocking every step of this cascade and by analyzing p-ERK1/2 expression by Immunoblot. As expected, phosphodiesterase inhibitor, 10 μM IBMX, strongly enhanced GUA-induced ERK phosphorylation [two-way ANOVA: IBMX effect *F*_(1,16)_ = 67.23, *P* < 0.0001]. Most importantly, NOS inhibitor, 1 mM L-NAME, completely prevented ERK1/2 phosphorylation elicited by GUA [two-way ANOVA: L-NAME effect *F*_(1,16)_ = 16.82, *P* < 0.0008]. The cell co-treatment with IBMX and L-NAME partially restored GUA-induced ERK phosphorylation, thus confirming the presence of other pathways converging on cGMP activation. The sGC inhibitor, 10 μM ODQ, likewise L-NAME, completely abrogated GUA-mediated ERK phosphorylation [two-way ANOVA: ODQ effect *F*_(1,16)_ = 25.33, *P* < 0.0001]. Of note, among the possible pathways involved in the PKB-ERK signaling, extracellular GUA stimulates the PI3K-PKB phosphorylation and NO production, thus evoking the downstream activation of sGC-cGMP-PKG pathway (**Figure 6**). Corroborating with these findings, PDE inhibitors have been shown to improve cognitive skills and memory formation in rodents (Reneerkens et al., 2009). The mechanism likely involves the induction of cAMP-protein kinase A (PKA)-cAMP responsive element-binding protein (CREB) and cGMP-PKG-CREB signaling pathways (Rutten et al., 2007), which are both associated with late-phase of Long-Term Potentiation (LTP).

### The Activation of the cAMP-Epac-CaMKII Pathway Influences NOS Activity

In an attempt to investigate whether the activation of collateral signaling pathway was able to affect the NO-sGC-cGMP-PKG-ERK cascade, we examined the possible role of cAMP-Epac-CaMKII pathway. CaMKII (Ca^2+^/calmodulin-dependent protein kinases II) is highly expressed in hippocamapal neurons and is involved in the glutamate-mediated LTP phase, wherein two major events occur: (i) Ca^2+^ enters the cell through NMDA channels and activates CaMKII that, in turn, recruits AMPA receptors to the plasma membrane; (ii) Ca^2+^ increases cAMP that activates ERK signaling (Giovannini, 2006; Miyamoto, 2006).

It has been recently proposed that extracellular cGMP regulates the glutamate-NO-cGMP pathway in a learning task, and this modulation resulted to be biphasic and relied on an inverse correlation between CaMKII and NOS activity (Moosavi et al., 2014; Cabrera-Pastor et al., 2016).

In our study, Immunoblot analysis of p-ERK showed that 2,5 μM 8-CPT-cAMP, an EPAC specific cAMP analog, inhibited GUA-induced ERK phosphorylation [two-way ANOVA: effect of 8-CPT-cAMP *F*_(1,16)_ = 18.16, *P* < 0.0006], although this result has to be further elucidated by using an EPAC-specific inhibitor. The inhibitor of CaMKII, 20 μM KN-93, caused an opposite effect and significantly enhanced p-ERK expression [two-way ANOVA: effect of KN-93 *F*_(1,16)_ = 30.80, *P* < 0.0001] (**Figure 6**). Therefore, we hypothesized that the functional interplay between NOS activity and CaMKII phosphorylation, observed in our experimental model, might have similar features to the above-mentioned glutamate-NO-cGMP pathway.

### GUA Enhances NO Production in SH-SY5Y Cells and Pre-treatment with L-NAME Prevents This Effect

**FIGURE 7:**
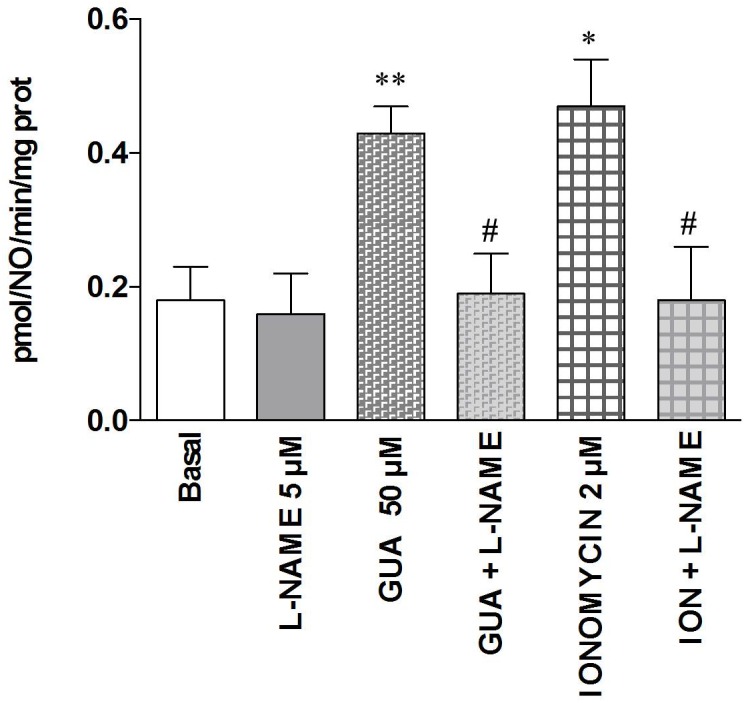
Guanine increases NO production in SH-SY5Y neuroblastoma cells. SH-SY5Y cells were treated for 30 min with 5 μM L-NAME or 50 μM GUA (alone or in combination), 2 μM ionomycin (ION), alone or in combination with 5 μM L-NAME. NOS activity was determined by the conversion of L-[3H]-arginine into L-[3H]-citrulline and expressed as pmol/NO/min/mg prot tot. Data were expressed as means ± SEM of four different experiments. Student’s *t*-test: ^∗^*P* < 0.05, ^∗∗^*P* < 0.01, vs. untreated cells (Basal); ^#^*P* < 0.05, vs. GUA-treated cells.

Finally, in order to compare the present data with those previously obtained *in vivo* (Giuliani et al., 2012), wherein L-NAME was able to prevent the GUA-mediated mnesic effect, we evaluated the effects of GUA on NO production. Cell exposure to 50 μM GUA for 30 min significantly increased NO production [two-way ANOVA: GUA effect *F*_(1,16)_ = 6.451, *P* < 0.0218, L-NAME effect *F*_(1,16)_ = 6.451, *P* < 0.0218 with interaction values between factors of *F*_(1,16)_ = 4.681, *P* = 0.0460] (**Figure 7**). A similar effect has been obtained when SH-SY5Y cells were challenged with 2 μM ionomycin, a calcium ionophore that induces NO production mainly via mobilization of intracellular Ca^2+^ [two-way ANOVA: GUA effect *F*_(1,16)_ = 5.523, *P* < 0.0319, ionomycin effect *F*_(1,16)_ = 5.523, *P* < 0.0319 with interaction values between factors of *F*_(1,16)_ = 4.190, *P* = 0.0575]. Cell pre-treatment (15 min before exposure to GUA or ionomycin) with 5 μM L-NAME, which *per se* did not affect the NO production of SH-SY5Y cells, strongly reduced the NO production induced by either GUA or ionomycin (**Figure 7**).

## Discussion

This work provides new insights on the transduction pathways involved in neuronal plasticity, in particular on the putative mechanism responsible of GUA-induced mnesic effects.

The major findings of the present study, performed in a model of neuronal-like cells (SH-SY5Y cells), are the following: (i) in resting condition, GUA is present in the intercellular milieu and derives from GTP breakdown, promoted by the activity of purine-converting enzymes (i.e., PNP and GDA) released in the culture medium; (ii) the inhibition of specific nucleoside and nucleobase transmembrane transporters enhances the extracellular levels of GUO, GUA and XAN; (iii) the addition of GUA to the culture medium caused a non-massive and dose-dependent production of ROS, and promotes the phosphorylation of ASK1, p38, JNK, PKC and PKB; (iv) extracellular GUA stimulates the PI3K-PKB phosphorylation and NO production, and activates the downstream sGC-cGMP-PKG-ERK pathway in a pertussis-toxin sensitive manner; (v) GUA-induced NO production is negatively modulated by the EPAC-cAMP-CAMKII pathway.

To better understand the results of this study, it has to be emphasized that, both *in vitro* and *in vivo* (Rathbone et al., 2008; Giuliani et al., 2012), the system of the extracellular guanine-based purines, together with a physiological and concerted activity of the enzymes regulating their metabolism (in particular PNP and GDA), is basically oriented to generate an higher amount of GUA rather than GUO or XAN; moreover, GUA stimulates the cGMP formation through NO production, which is known to modulate a broad range of effects in the CNS, such as neuronal development and synaptic plasticity (Prast and Philippu, 2001).

The evidence of the involvement of NO-cGMP pathway in some forms of learning and memory has been worked out by many authors (Erceg et al., 2005; Piedrafita et al., 2007; Ben Aissa et al., 2016; Cabrera-Pastor et al., 2016; Zhou et al., 2017). In several brain areas (i.e., hippocampus, cerebellum and striatum), increased levels of cGMP seemed to improve learning and memory consolidation, mainly during early stages of memory formation (immediately after training). This data is confirmed by full amnesia for inhibitory avoidance task when rats were treated with LY 83583, a soluble guanylate cyclase inhibitor, immediately after training (Bernabeu et al., 1997). However, not all signal transduction pathways, leading to cGMP formation, cause beneficial effects on cognitive functions. For example, cGMP has been found to mediate the stimulation of dendritic number and branching as well as the neurite elongation. This event likely occurs as a consequence of an interplay between cGMP and the activity of extrinsic/external factors such as neurotrophins (Tian et al., 2017) that, usually, stimulate cAMP formation. Cyclic nucleotides are differently involved in the memory consolidation process, since it has been reported that cGMP regulates the early consolidation phase, whereas cAMP is implicated in late processes of memory formation (Izquierdo et al., 2006; Rutten et al., 2007; Bollen et al., 2014). In this functional interplay, a significant role seems to be played by the guanine-based purines, in particular by GUO and GUA. Indeed, several studies reported that GUO causes memory impairment (Roesler et al., 2000; Vinadé et al., 2003, 2004) but, at the same time and sinergistically with NGF, it promotes neurite outgrowth in PC12 cells via activation of heme-oxygenase and cGMP formation (Gysbers and Rathbone, 1996; Bau et al., 2005; Tomaselli et al., 2005). Cell exposure to neurotrophic factors such as NGF, for more than 24 h, promotes the phosphorylation of PKC-Ras-MAPK (Kumar et al., 2005) and, in turn, increases the mRNA levels of GDA, responsible of GUA degradation (Rathbone et al., 1999). This enzyme favors dendrite branching and elongation by directly binding tubulin heterodimers, thus promoting the microtubule assembly via cytoskeletal rearrangement (Akum et al., 2004). Therefore, the synergistic activity of neurotrophins with agents promoting neuritogenesis, such as GUO, explains why the neurogenic activity of GUO, despite being linked to an increased cGMP formation, is associated with an amnesic effect. Conversely, GUA promotes the early stages of memory formation in rats (Giuliani et al., 2012) and its effect seems to be linked to the NO-cGMP signaling pathway. In line with it, we observed that L-NAME prevented memory consolidation caused by the administration of GUA *in vivo*, in a model of passive avoidance task (Giuliani et al., 2012). In the same study, the administration of GUA, 15 min before treatment with L-NAME, prevented the amnesic effect of the NOS inhibitor.

The involvement of the NO-cGMP-PKG-ERK signaling pathway in synaptic plasticity has been extensively reported (Chien et al., 2008; Ota et al., 2010; Bartus et al., 2013; Matsumoto et al., 2016). However, the role of NO in each step of learning appears controversial and it might be task-dependent. ERK cascade is positively implicated in the development of fear conditioning, conditioned taste aversion memory, spatial memory, step-down inhibitory avoidance and object recognition memory (Giovannini, 2006; Chen et al., 2017; Vithayathil et al., 2017). In our study, this pathway resulted to be activated upon cell exposure to GUA. Specifically, extracellular GUA stimulated the PI3K-PKB phosphorylation and NO production, and activated the downstream sGC-cGMP-PKG-ERK pathway. It is feasible that GUA-induced ROS production, with the subsequent phosphorylation of ASK1, p38 and JNK, may contribute to the cGMP formation trough other pathways than NO, as confirmed by our data, wherein, in cells pre-treated with L-NAME, the inhibition of phosphodiesterases caused a limited increase in GUA-induced ERK1/2 phosphorylation.

Noteworthy, if the role of cGMP in learning and memory is important, much more valuable is that its mnesic effect is mediated by NO production. Indeed, the increase of the intracellular levels of cGMP induced by GUO, through the activation of HEME-oxygenase and independent of the NO production, is important for the dendrite and neurite outgrowth but it is associated with amnesia (Vinadé et al., 2004; Bau et al., 2005).

We, then, took into account the existence of a more complex network, wherein the NO production is eventually under the control of other molecular mechanisms and, among them, we investigated the EPAC-CAMKII system. CaMKII has a prominent role in memory formation (LTM) (Tan, 2007; Takao et al., 2010; Giese and Mizuno, 2013; Nakamura et al., 2017). For this purpose, we took advantage of a recent study, where they showed that the activation of CAMKII, upon stimulation of NMDA receptors, inhibits the production of NO that functions as a retrograde signal able to modulate the glutamatergic system (Cabrera-Pastor et al., 2016). Noteworthy, in our model, the activity of GUA was similar to that of glutamate, since we observed that the stimulation of the EPAC-CAMKII pathway inhibited the NO-mediated phosphorylation of ERK1/2 induced by GUA. The blockage of this pathway by using a CaMKII inhibitor, KN-93, amplified GUA effect. Consistent with it, two endogenous CaMKII inhibitors (CaMK2N1 and CaMK2N2) have been shown to prevent memory loss after retrieval (Vigil et al., 2017). However, it should be pointed out that the influence exerted by CaMKII on the phosphorylation of ERK is not univocal. Indeed, it has been reported that: (i) in vascular smooth muscle cells the CaMKII inhibitor KN-93 caused an H_2_O_2_-mediated reduction of ERK1/2 and PKB phosphorylation (Robison et al., 2007; Bouallegue et al., 2009); (ii) the presynaptic injection of a CaMKII inhibitor blocked LTP and neurotransmitter release induced by either the NO donor or the PKG activator (Feil and Kleppisch, 2008).

Finally, we speculated that the extracellular effect of GUA may be mediated by a membrane receptor at present not well identified. In this study, cell treatment with pertussis toxin strongly reduced the GUA-evoked ERK1/2 phosphorylation, thus indicating that this putative new GUA receptor is likely coupled with Gi/o proteins.

Guanine-based purines, in particular GUO, seems to bind to metabotropic receptors and many of their effects are mediated through G-protein-dependent signaling pathways; it has been shown, for instance, that the pertussis toxin-mediated inhibition of Gi/Go-protein reverses some of the effects of GUO on cell viability and glutamate uptake in hippocampal slices (Traversa et al., 2003; Dal-Cim et al., 2012, 2013).

Several years ago, our group found a specific [3H]-guanosine binding site in rat brain membranes, compatible with an unknown G protein-coupled receptor (Traversa et al., 2002, 2003; Di Liberto et al., 2012; Grillo et al., 2012). In order to provide insight into the characteristics of the binding site, we evaluated the relative abilities of purine analogs to displace [3H]GUO. Binding data revealed that all the adenine-based purines as well as GTP, GDP and GMP were ineffective in displacing [3H]GUO. The 6-Thio-GUO or 6-keto-GUO derivatives resulted to be as effective as GUO in displacing [3H]GUO. On the contrary, the binding affinity was strongly reduced when the 6-amino or 2-amino derivatives were assayed.

These findings seem to be compatible with a membrane binding site, expressed in the rat brain, which, in addition to GUO, may interact with GUA, although with a lower affinity. At present, we are carrying out a study to individuate and functionally characterize this new putative receptor.

At the same time, it cannot be excluded an interaction of GUA with another receptor functionally different from G protein-coupled receptors. Indeed, it is well known that compounds that are structurally very similar to GUO and GUA represent the main agonists of several Toll-like receptors (i.e., TLR9, 7 and 8) (Lee et al., 2003; Yu et al., 2017).

In this context, it has been recently reported that the stimulation of some subtypes of Toll-Like receptors (TLR9, 7 and 8) in microglial cells leads to cognitive improvements and ameliorates the vascular amyloid pathology in triple transgenic mice expressing human Swedish K670N/M671L and vasculotropic Dutch/Iowa E693Q/D694N mutations and exhibiting early cerebral microvascular accumulation of Aβ (Scholtzova et al., 2017). Interestingly, some guanine-based purines and their modified derivatives have been recently recognized as endogenous ligands for TLRs, especially 7 and 9 subtypes (Shibata et al., 2016; Abdul-Cader et al., 2017). In this regard, in order to eliminate the effects potentially mediated by TLRs, in our study we used cultured SH-SY5Y cells, wherein the expression of TLR 7/8 and 9 is not reported in literature.

## Conclusion

Targeting NO-cGMP-PKG-ERK signaling pathway may represent an interesting approach for the development of new drugs in the treatment of memory dysfunctions occurring in neurodegenerative and psychiatric diseases, among others Alzheimer’s disease and dementia. Initial promising findings in this direction have been reported regarding the use of PDE inhibitors (Kumar and Singh, 2017; Prickaerts et al., 2017) or sGC stimulator (Montfort et al., 2017) in the treatment of neuroinflammatory and neuropathological conditions.

It is plausible to expect that, beyond the above-mentioned guanine-derivatives, the administration of GUA itself (Giuliani et al., 2012), due to its long half-life *in vivo*, may elicit molecular changes that underlie synaptic alterations and memory formation through a putative receptor that could represent a new pharmacological target. This may serve the purpose of avoiding a major challenge, that is to discriminate the numerous downstream effectors ensuing the activation of NO-cGMP-ERK signaling pathway, thus bypassing the sophisticated network of different and multifunctional protein kinases.

## Author Contributions

All authors listed have made a substantial, direct and intellectual contribution to the work, and approved it for publication.

## Conflict of Interest Statement

The authors declare that the research was conducted in the absence of any commercial or financial relationships that could be construed as a potential conflict of interest.
